# Treatment of mycophenolate-resistant immune-related organizing pneumonia with infliximab

**DOI:** 10.1186/s40425-018-0400-4

**Published:** 2018-09-03

**Authors:** Guacimara Ortega Sanchez, Kathleen Jahn, Spasenija Savic, Alfred Zippelius, Heinz Läubli

**Affiliations:** 1 Department of Internal Medicine, Division of Medical Oncology, University Hospital Basel, Basel, Switzerland; 2 Division of Pneumology and Pulmonary Cell Research, University Hospital Basel, Basel, Switzerland; 3 Institute of Pathology, University Hospital Basel, Basel, Switzerland; 4 Department of Biomedicine, Cancer Immunology Laboratory, University Hospital Basel, Basel, Switzerland; 5 Medical Oncology and Cancer Immunology, University Hospital Basel, Petersgraben 4, 4031 Basel, Switzerland

**Keywords:** Immune checkpoint inhibitor, Cancer immunotherapy, Pneumonitis, Lung, Immune-related adverse event

## Abstract

**Background:**

The development of pulmonary immune-related adverse events (irAEs) in patients undergoing PD-(L)1 targeted checkpoint inhibitors are rare, but may be life-threatening. While many published articles and guidelines are focusing on the presentation and upfront treatment of pulmonary irAEs, the strategy in patients with late-onset pneumonia that are resistant to commonly used immunosuppressive drugs remains unclear.

**Case presentation:**

Here, we report the successful treatment of a mycophenolate-resistant organizing pneumonia (OP) with infliximab in a patient with metastatic melanoma after PD-1 blockade. The patient received two years of PD-1 targeted immunotherapy when he developed multiple nodular lung lesions mimicking a metastatic progression. However, wedge resection of these lesions showed defined areas of OP, which responded well to corticosteroids. Upon tapering, new foci of OP developed which were resistant to high-dose steroids and mycophenolate treatment. The TNFα antagonist infliximab led to a rapid and durable regression of the inflammatory lesions.

**Conclusion:**

This case describes a not well-studied situation, in which a mycophenolate-resistant PD-1 blocker-associated pneumonitis was successfully treated with a TNFα neutralizing antibody. The outcome of this case suggests that infliximab might be the preferable option compared to classical immunosuppressants in the case of steroid-resistant/−dependent late onset pulmonary irAEs.

## Background

Blocking antibodies that target the immune checkpoint PD-(L)1 have led to durable remissions in various cancers including but not limited to melanoma, non-small cell lung cancer (NSCLC), bladder cancer and renal cell carcinoma [1–3]. Although PD-(L)1 targeted checkpoint inhibitors are most often well tolerated, 10–15% patients develop severe immune-related adverse events (irAEs) [4–8]. In addition, combination immunotherapies including PD-(L)1 and CTLA-4 targeted therapies have been approved and show an increased frequency of irAEs [9, 10]. Affections of the lung with irAEs are among the most dangerous and also most heterogenous side effects of immune checkpoint inhibitors [7, 8, 11, 12]. A recent analysis of 915 patients showed a frequency of 5% (43 patients) in patients with PD-L(1) targeted monotherapy [12]. While guidelines for the treatment of pulmonary irAEs have been developed and help to manage these side effects ([4, 6, 8]; NCCN guidelines), the use of the optimal immunosuppressant in patients not or insufficiently responding to steroids remains less clear. Here, we describe a case with late-onset pulmonary irAE presenting as an organizing pneumonia (OP) that developed during PD-1 targeted checkpoint blockade with a corticosteroid dependency and resistance to classical immunosuppressants. We also summarize the current evidence for treatment strategies of steroid-resistant/−dependent pulmonary irAEs.

## Case presentation

**Fig. 1:**
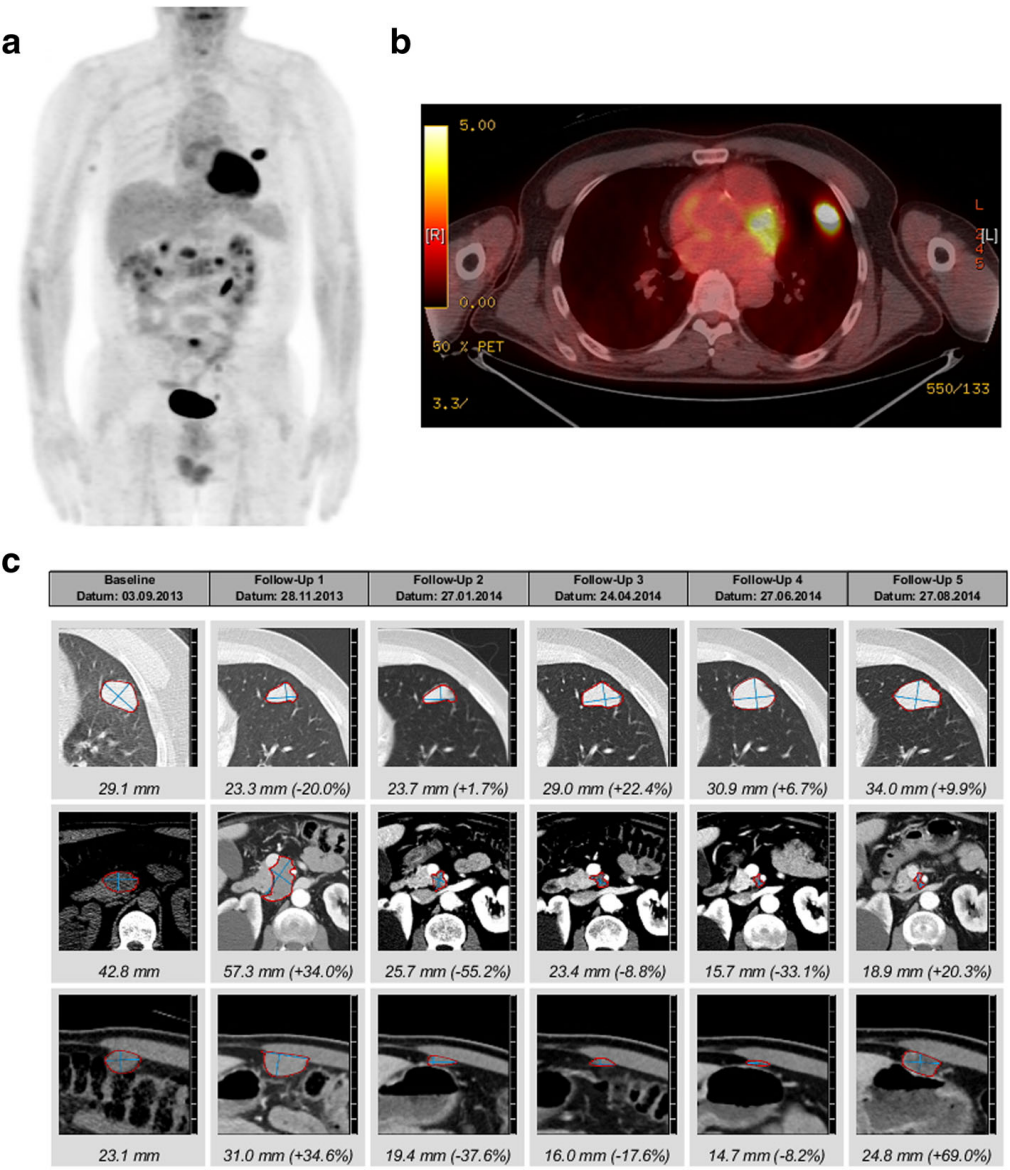
Initial treatment of metastatic melanoma. **a** ^18^FDG-PET-CT scan of the patient when the metastatic disease was diagnosed. **b** ^18^FDG-positive lung lesion at the initial diagnosis of metastatic BRAF mutated melanoma. **c** Initial treatment response to BRAF and MEK inhibitors and to pembrolizumab over time

**Fig. 2:**
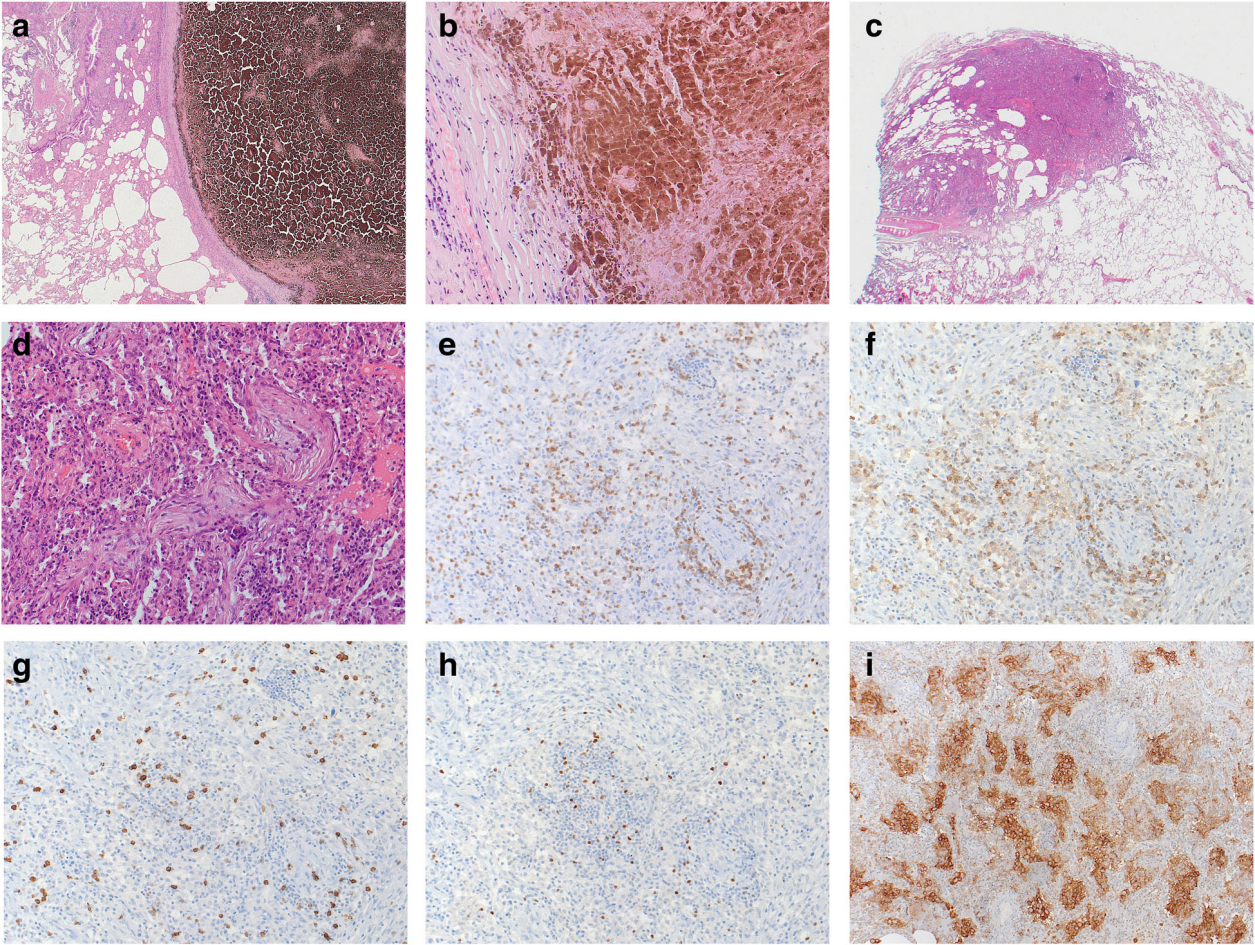
Histology of metastasis and organizing pneumonia. **a**, **b** Wedge resection of the lung after immunotherapy. The melanoma metastasis shows excellent response to treatment with extensive regressive necrosis. (HE, original magnification 25× and 200×, respectively). Here only melanin pigment and necrosis with lack of vital tumor cells are present. **c**, **d** Distant to the necrotic metastasis circumscribed areas of immunotherapy-induced organizing pneumonia with intraalveolar fibromyxoid proliferations and mild lymphoplasmacytic inflammation (HE original magnification 12,5× and 200×, respectively). (**e**) Immunhistochemical analysis of CD3^+^ cells in inflammatory lesions (200× magnification). **f, g** Staining of CD4^+^ (**f**) and CD8^+^ (**g**) cells (200× magnification). **h** Staining for FOXP3^+^ regulatory T cells in inflammatory lesions (200× magnification). **i** Immunostaining for PD-L1 in inflammatory lesions (100× magnification, SP-263 clone)

**Fig. 3:**
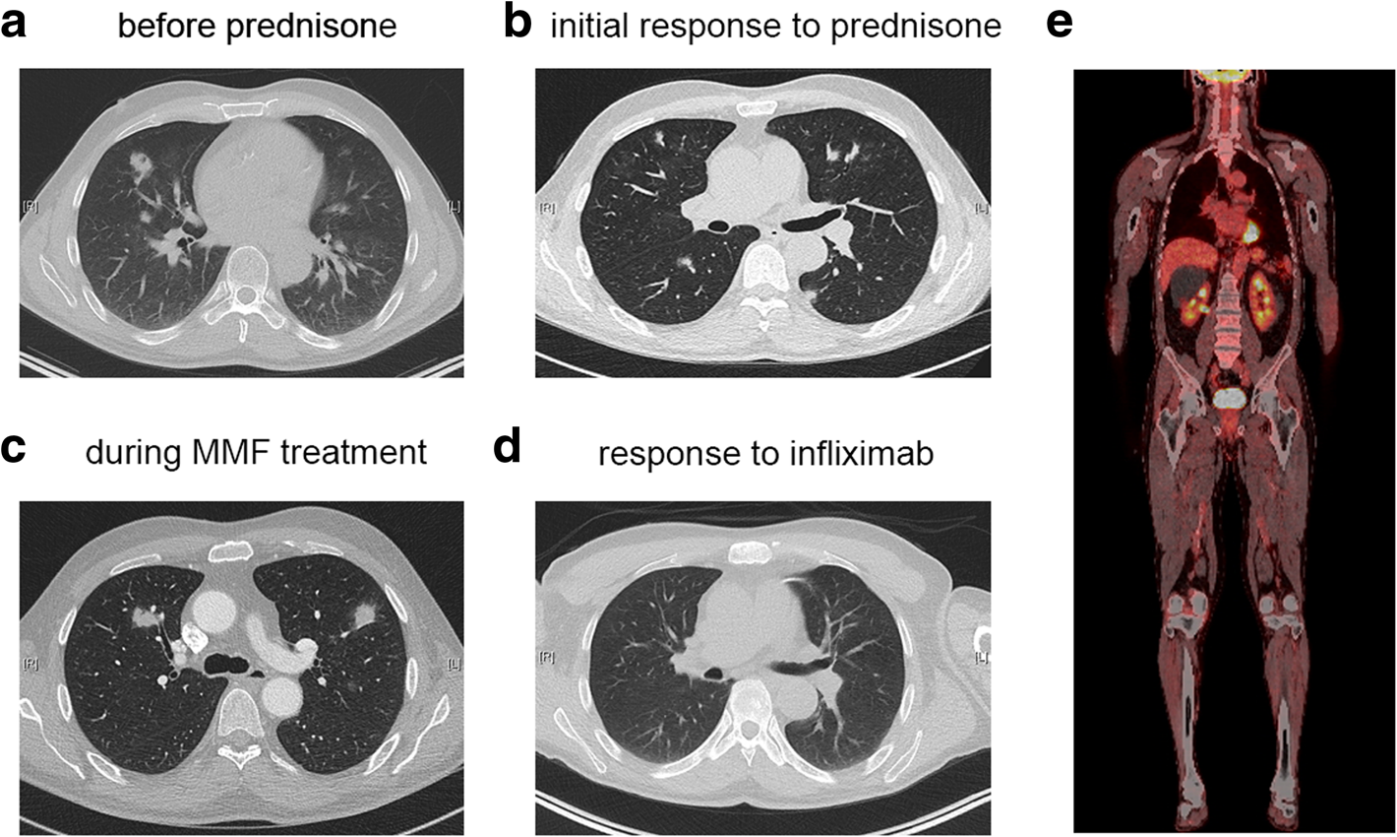
Response of pulmonary inflammatory lesions to immune suppressive therapy. **a** CT-scan at the time point when the inflammatory lesions were first detected after 2 years of pembrolizumab therapy. **b** Initial response to corticosteroid therapy (1 mg/kg prednisone). Pulmonary inflammatory lesions were clearly regressing after initial therapy. **c** CT-scan acquired during immune suppression with mycophenolate and after tapering of prednisone (before start infliximab). **d** CT-scan after 6 months of infliximab therapy. No inflammatory lesions can be observed anymore. **e** ^18^FDG-PET-CT scan after the infliximab therapy was stopped. A durable remission in terms of the melanoma and the pneumopathy was found

We report a 75-year old man with stage IV BRAF V600E mutated malignant melanoma. On his initial ^18^fluoro-deoxy-glucose (FDG) positron emission tomography computed tomography (PET-CT) scan, he presented with multiple bilateral pulmonary nodules, bone and cutaneous lesions, peritoneal metastases and a lesion at the head of the pancreas (Fig. 1a and b). A palliative combination therapy with a BRAF- (dabrafenib 2x 150 mg) and MEK inhibitor (trametinib 2 mg) was started. Six weeks later a CT-scan revealed a partial remission of the lung, bone and cutaneous lesions but a progression of the lesion in the pancreas. A fine needle aspiration of the pancreatic lesion confirmed metastasis of the melanoma. This metastasis was irradiated and the combination targeted therapy continued. Eight months later, a progression with several new pulmonary lesions and peritoneal metastasis (Fig. 1) was observed and a second line therapy with the CTLA4 inhibitor ipilimumab (3 mg/kg) started. After 2 cycles, a disease progression (Fig. 1c) prompted a third line therapy with the PD-1 inhibitor pembrolizumab and radiotherapy of a myocardial metastasis. After the start of pembrolizumab, the condition of the patient rapidly improved and the patient achieved a good partial remission (Fig. 1). At 24 months under pembrolizumab a routine CT-scan showed multiple bilateral part solid lung lesions in the upper parts of the lung. At that stage, the patient reported NYHA II dyspnea. Endoscopically the tracheobronchial system was unremarkable. Bronchoalveolar lavage (BAL) demonstrated only a slight lymphocytosis of 13% lymphocytes without signs of pulmonary infection (negative microbiological cultures and PCR for viral pathogens). A transbronchial lung biopsy showed only normal lung morphology. Since it was unclear if the new lesions were metastases, we decided to surgically obtain a histological specimen. Wedge resection of several nodular lesions of the lung was therefore performed. Surprisingly, only one pulmonary lesion represented a melanoma metastasis with almost complete regressive necrosis as a sign of excellent response to treatment. The other lesions distant to the metastasis represented circumscribed areas of OP (Fig. 2). We found a strong CD3^+^ cell infiltration of the inflammatory lesion with predominantly CD4^+^ cells over CD8^+^ cells (Fig. 2e-g). Also, a several FOXP3^+^ cells were found (Fig. 2h). Interestingly, clusters of PD-L1 positive macrophages were seen (Fig. 2i, SP-263 clone). A therapy with corticosteroids according to current guidelines for grade I-II pulmonary irAE was initiated with prednisone of 1 mg/kg and stopping of pembrolizumab. After a rapid regression of pulmonary lesions, the corticosteroid dose was tapered to 10 mg daily during a time period of 7 weeks. At this point, the patient presented with dyspnoea NYHA II and mainly thoracic pain. A CT revealed no lung embolism, but a progression of the bilateral part solid lung lesions corresponding to OP (Fig. 3a and b). With the OP relapse, a course of antibiotics and corticosteroids 50 mg daily was initiated with a rapid clinical and radiological improvement. A reduction of the corticosteroids by 10 mg every 4 weeks was recommended as performed for OP in routine pneumological practice. However, as soon as the doses of prednisone were lower than 20 mg daily a clinical and radiological worsening was observed. For dose reduction/ steroid sparing we decided to establish a combined immunosuppressive therapy consisting of mycophenolate mofetil (Cellcept) twice daily when the dose of prednisone 20 mg daily was reached again. After 4 weeks, the patients relapsed again with dyspnoea and new pulmonary lesions on the CT-scan (Fig. 3c). Because of the insufficient response to mycophenolate the TNFα-blocking agent infliximab was started. Initial dosing with 5 mg/kg was given for 3 doses and then a maintenance therapy was installed (100 mg). This led to a significant improvement after 4 months of therapy and corticosteroids could be completely stopped. The patient developed again dyspnoea at exertion, but a CT-scan showed no lung lesions or any direct cause for the dyspnoea (Fig. 3e). A cardiac stress test demonstrated some myocardial perfusion deficit and a coronary angiogram a high-grade stenosis of the left anterior descending (LAD) artery, which could be dilated and stented with a drug-eluting stent. After 6 months, infliximab was stopped and the patient is currently in an enduring very good partial remission for his melanoma (Fig. 3e).

## Discussion

Immune checkpoint inhibitors are unequivocally one of the most important breakthroughs in cancer therapy in the past 10 years [2, 3, 13]. Immune system activation leads to rejection of cancer cells but may be harmful to healthy tissues [14]. Common irAEs include cutaneous, gastrointestinal, hepatic, pulmonary, and endocrine events [4–7]. Among them, immune-related pneumonitis has been reported to be a relatively uncommon but serious and potentially life-threatening irAE and has resulted in pneumonitis-related death in several Phase I trials [15–17]. Topalian et and colleagues reported several cases of pneumonitis with different findings ranging from isolated radiographic abnormalities to progressive, diffuse infiltrates associated with clinical symptoms which resulted in 3 deaths [15]. Pneumonitis-though a non-specific term- is defined as a non-infectious focal or diffuse reaction of the lung parenchyma, and includes different histological findings, e.g. sarcoid-like reaction, lymphocytic alveolitis, OP or even diffuse alveolar damage. Its incidence in studies with anti–PD-(L)1 mAbs has ranged from 0 to 10% [12, 18, 19]. Drug-related pneumonitis can also occur with chemotherapy (docetaxel, gemcitabine, bleomycin), targeted therapy (e.g. EGFR inhibitors, mTOR inhibitors), and radiation therapy. Its clinical characteristics are unspecific including non-productive cough, tachypnea and dyspnea on exertion and less often tachycardia, cyanosis, fatigue and occasional fever and chills. The presentation of pneumonitis is complicated with regard to diagnosis and unpredictable, and the disease tends to occur later than other irAEs [4–7, 12]. BAL can help to elucidate the underlying reaction of the lung parenchyma, for example in lymphocytic alveolitis. There are no criteria to differentiate drug-induced pneumonitis from other types of pneumonitis, nor are there criteria for assessing disease progression, which is important for determining treatment plans. Differential diagnoses have often to be considered including infections and also cancer  progression. In particular in patients with primary pulmonary lesions or an underlying lung disorder, the diagnosis can be even more difficult. In this case, diagnosis was only made after thoracic surgery was performed. In unclear cases like the one presented, we always aim to obtain a histological diagnosis. Pulmonary irAEs – early and late onset – are graded based on the severity of its associated radiographic alterations and clinical symptoms. Grade 1 pneumonitis presents as radiographic alterations without respiratory discomfort, whereas grade 2 pneumonitis is characterized by low-intensity clinical symptoms. Grade 3–4 pneumonitis causes severe clinical symptoms, such as dyspnea, cough, and hypoxia [4–7]. Our patient presented initially with grade 2 pneumonitis.

The rate of grade 3/4 pneumonitis associated with nivolumab treatment was ≤1% in phase 3 non-small-cell lung cancer studies CheckMate-017, and CheckMate-057, with no deaths reported due to pneumonitis [18, 19]. Similar rates were seen in first line trials with nivolumab or pembrolizumab [20, 21]. Symptomatic pneumonitis is quite rare with ipilimumab monotherapy, and diffuse, grade 3/4 pulmonary toxicity is one of the irAEs that distinguishes anti-PD-(L)1 and anti-CTLA-4 immune checkpoint inhibitors [9, 22]. However, combination immunotherapy with ipilimumab and nivolumab for melanoma in the CeckMate 067 increased the risk for immune-related pneumopathies [9]. In the CheckMate-214 trial of ipilimumab in combination with nivolumab for patients with metastatic RCC, one patient even died because of an immune-related pneumonitis [10]. Nishino and colleagues reviewed the frequency across different trials [23]. The odds in patients with melanoma and RCC that received combination therapy with ipilimumab and nivolumab was significantly increased (OR, 2.04, 95% CI 1.69–2.50, *p* < 0.001) [23]. Khunger and colleagues also performed a systematic review and meta-analysis of trials in the literature to evaluate the incidence pneumonitis with use of PD-(L)1 inhibitors in non-small cell lung cancer [24]. Nineteen trials (12 with PD-1 inhibitors [*n* = 3232] and 7 with PD-L1 inhibitors [*n* = 1806]) were identified. PD-1 inhibitors were found to have statistically significant higher incidence of any grade pneumonitis compared with PD-L1 inhibitors (3.6%; 95% CI, 2.4–4.9% vs 1.3%; 95% CI, 0.8–1.9%, *p* = .001). PD-1 inhibitors were also associated with higher incidence of grade 3 or 4 pneumonitis (1.1%; 95% CI, 0.6–1.7% vs 0.4%; 95% CI, 0–0.8%; *p* = .02). Treatment naive patients had higher incidence of grade 1 through 4 pneumonitis compared with previously treated patients (4.3%; 95% CI, 2.4–6.3% vs 2.8%; 95% CI, 1.7–4%; *p* = .03). Naidoo et al. described clinical, radiologic, and pathologic features and management of 43 cases of pneumonitis as a result of anti–PD-1/PD-L1 mAbs from two separate institutions (Memorial Sloan Kettering Cancer Center: advanced solid cancers, 2009 to 2014, and Melanoma Institute of Australia: melanomas only) [12]. The overall incidence of pneumonitis was 5% (43 of 915 patients). Incidence was similar among patients with melanoma and NSCLC for monotherapy (3.6% vs 3.3%). Pneumonitis occurred irrespective of line of therapy in which immunotherapy was received (first line, 32%; second line, 40%; third line or more, 28%). All patients with grade 2 pneumonitis were treated initially with oral/intravenous corticosteroids (*n* = 14). Another overview of patterns showed also quite some heterogeneity [25]. Among 170 patients undergoing PD-(L)1 blocking therapy, 20 patients developed any grade of pneumonitis [25]. The most common radiographic pattern was consistent with OP similar as in our case.

Guidelines for the treatment of immune-related pneumopathy that developed during checkpoint blockade in the case of steroid-refractory or steroid-dependent disease exist ([4–7]; NCCN guidelines). Several immunosuppressants are suggested including mycophenolate, or cyclophosphamide. Cases of pneumonitis with treatment strategies have been described in the literature. In one case, an immune-related pneumonitis caused by PD-1 inhibitor pembrolizumab in a patient with advanced esophageal carcinoma was described [26]. After six cycles of pembrolizumab (3 mg/kg every 3 weeks), the CT-scan showed diffuse ground glass opacity and consolidation in both lungs. Cellular interstitial pneumonitis was confirmed by pathological examination. Patient’s symptoms were alleviated after corticosteroid therapy. The pneumonitis reoccurred twice after stopping or tapering steroids quickly and could also be controlled by using steroids again (steroid dependency). No specifics were given of alternative immunosuppressants [26]. Three cases with pneumonitis were reported in patients that have received radiotherapy prior to PD-1 blockade [27]. A series of 3 cases reported 1 patient who received nivolumab and ipilimumab sequentially, a second patient was treated with nivolumab and a third patient with ipilimumab before starting nivolumab [28]. The onset of pneumonitis occurred 7.4 to 24.3 months after the initiation of therapy. It is important to be aware that patients can develop this severe side effect even years after the initiation of PD-(L)1 blockade. CT imaging revealed findings seen in interstitial pneumonias. These conditions were CT-morphologically classified as acute interstitial pneumonia–acute respiratory distress syndrome (diffuse alveolar damage). Two patients were admitted to the intensive care unit and received intravenous antibiotic agents, glucocorticoids, and infliximab. Patient 1 required intubation, and his condition improved over the course of 10 weeks. Patient 2 died 4 weeks after the diagnosis of pneumonitis. Patient 3 had ground-glass opacities and reticular opacities indicative of nonspecific interstitial pneumonia. He discontinued nivolumab for 8 weeks and received oral glucocorticoids as an outpatient, and the pneumonitis resolved. In the cases reviewed by Naidoo et al., all patients with grade 3 or higher pneumonitis received oral/intravenous corticosteroids initially (*n* = 12), five (42%) of whom required additional immunosuppression (three with infliximab and two with both infliximab plus cyclophosphamide) [12]. For most patients who began oral corticosteroids, this was the maximum immunosuppression used (14 of 17 [82%]), with a median starting dose of prednisone of 50 mg (range, 20 to 80 mg) and median duration of corticosteroid treatment of 68 days (range, 20 to 154 days). In another cohort, 3 out 20 patients with pneumonitis were treated with infliximab [25]. In one patient, a combination treatment with cyclophosphamide and infliximab was needed. An overview of published cases with pneumonitis and infliximab treatment can be seen in Table 1. According to current evidence and guidelines ([4, 6, 7];NCCN guidelines), we treated the patient initially with 1 mg/kg prednisone for grade 2 late onset immune-related pneumonitis. The patient became steroid-refractory and the immune suppression was extended by adding the classical immunosuppressant mycophenolate mofetil. It remains unclear which type of additional immunosuppressants is preferable in this situation. The late onset organizing pneumonia was not controlled and a further therapy with infliximab was started with rapidly improving pulmonary lesions. This outcome suggests that in the case of late onset pneumonitis during immune checkpoint blockade, TNFα inhibitors might be preferable compared to classical immunosuppressants, although more studies are needed to confirm this.

**Table 1:** Overview of published pneumonitis cases upon PD-(L)1 blockade that received a treatment with infliximab

Publication	Number of Patients (%)	Patients with pneumonitis (%)	Treatment
Chen et al, [26]	1	1	Corticosteroids
Lu et al, [27]	3	3	Corticosteroids
Nishino et al, [28]	3	3	Corticosteroids, 2 patients with infliximab
Naidoo et al, [12]	915	43 (5%) 12 Grade 3–4 (1.3%)	Corticosteroids, 5 patients with infliximab
Nishino et al, [25]	170	20 (11.8%)	Corticosteroids, 3 patients with infliximab

## Conclusion

We report here the first case of steroid- and mycophenolate mofetil-resistant pneumonitis that presented as OP as a complication of PD-1-directed immunotherapy for melanoma. Immune-related pneumopathies are an uncommon but potentially serious toxicity that occurs in approximately 5% of patients who receive anti–PD-(L)1 mAbs. Most cases are mild and managed successfully with favorable outcomes. However, worsening pneumonitis may develop in a subset of patients despite additional immunosuppression, and they may suffer also from the immunosuppressive consequences of pneumonitis treatment. Infliximab can be used in steroid-refractory immune-related pneumopathies and might be preferable compared to other classical immunosuppressants. However, improvements in the treatment and understanding of the biology of immune-related pneumopathy are needed to optimize management.

## Abbreviations

CT: Computed tomography
irAE: immune-related Adverse event
mAb: monoclonal antibody
MRI: Magnetic resonance imaging
PD-1: Programmed Death-1
PD-L1 or –L2: Programmed Death Ligand-1 or − 2
TNFα: Tumor Necrosis Factor α
